# Interleukin 1 is a key driver of inflammatory bowel disease-demonstration in a murine IL-1Ra knockout model

**DOI:** 10.18632/oncotarget.26894

**Published:** 2019-05-28

**Authors:** Rasha H. Dosh, Nicola Jordan-Mahy, Christopher Sammon, Christine Le Maitre

**Affiliations:** ^1^ Biomolecular Sciences Research Centre, Sheffield Hallam University, Sheffield, UK; ^2^ Materials and Engineering Research Institute, Sheffield Hallam University, Sheffield, UK; ^3^ Department of Anatomy and Histology, Faculty of Medicine, University of Kufa, Kufa, Iraq

**Keywords:** IL-1Ra deficient, inflammatory bowel disease, pro-inflammatory cytokines, matrix-degrading enzymes, intestinal enzymes

## Abstract

Interleukin 1 (IL-1) is an important mediator of inflammation and tissue damage in inflammatory bowel disease (IBD). The balance between IL-1 and IL-1Ra as a natural inhibitor plays a vital role in a variety of diseases. Here, we investigated whether changes seen during IBD are induced spontaneously in mice lacking a functional *IL-1rn* gene. Histological staining was performed on the jejunum and ileum of BALB/c *IL-1rn^+/+^* and *IL-1rn^-/-^* mice to characterize crypt-villus height, villus width, and number of goblet cells per villus. Pro-inflammatory cytokines, immune cell infiltration and matrix-degrading enzymes, together with the production of intestinal enzymes and the integrity of tight and adherent junction proteins were determined using immunohistochemistry. In the small intestine of BALB/c *IL-1rn^-/-^* mice the villus heights were significantly reduced; and in the ileum this was accompanied by a decrease in villi width. There was also an increase in goblet cell number and mucin production compared to wild-type mice. IL-1α and IL-1β immunopositivity were increased, whilst IL-1R1 expression was decreased in *IL-1rn^-/-^* mice. IL-15 and TNFα were also increased in older *IL-1rn^-/-^* mice. Increased polymorphonuclear and macrophage infiltration were seen in *IL-1rn^-/-^* mice, whilst expression of matrix-degrading enzymes and digestive enzymes were unchanged, except for dipeptidyl peptidase IV which was increased in younger *IL-1rn^-/-^* mice compared to wild type mice. The expression of tight and adhesion junctions were also dramatically decreased in *IL-1rn^-/-^* mice. In conclusion, *IL-1rn^-/-^* mice developed spontaneous abnormalities which displayed features associated with IBD, demonstrating a clear role for IL-1 in IBD.

## INTRODUCTION

Inflammatory bowel disease (IBD) is a chronic autoimmune disease characterised by inflammation of the gastrointestinal tract and can be divided into two main types. Crohn’s disease (CD), which affects any part of the gastrointestinal tract especially the terminal ileum and colon; and ulcerative colitis (UC) which affects the rectum and colon [1]. Although the pathogenesis of IBD is not fully understood, IBD is thought to be caused by an imbalance between pro- and anti-inflammatory cytokines in local tissues which leads to inflammation and malfunction of the barriers in the intestinal tissue.

Interleukin 1 (IL-1) is a key mediator of innate immunity and inflammation which results in tissue damage in IBD. An imbalance between IL-1 and IL-1Ra has been shown in the inflamed mucosa of patients with IBD where the levels of IL-1 and IL-Ra were increased, but importantly the ratio of IL-1Ra to IL-1 was significantly decreased compared with controls [1, 2]. During Crohn's disease, IL-1β is significantly raised and there is a positive correlation between the severity of mucosal inflammation and the levels of IL-1β [3]. It can also result in apoptosis of epithelial cells causing tissue damage and barrier dysfunction. Subsequently, the role of IL-1Ra in disease progression has been studied in various experimental animal models of IBD [4–6].

In the SAMP/YiT experimental mice model, there was an increase in Th-1 activity which mediates intestinal inflammation, and there was a spontaneous appearance of chronic ileitis which was similar in appearance to Crohn's disease due to increased intestinal paracellular permeability [7, 8]. This chronic ileitis is characterised by intestinal inflammation, having mononuclear and polymorphonuclear infiltration of the lamina propria; plus hyperplasia of paneth and goblet cells [9].

Although studies have reported that an imbalance between IL-1 and IL-1Ra contributes to inflammation in the large intestine in patients of IBD and in experimental animal models [1, 10, 11], whether this is a causative factor is unclear. Here, the histological changes in the small intestine of IL-1Ra knockout mice were investigated to assess the expression of pro-inflammatory cytokines, infiltration of immune cells, matrix-degrading enzymes, junctional proteins, and digestive enzymes in the small intestine of IL-1Ra knockout mice compared to wild-type mice in two age groups in order to determine whether features associated with IBD could be induced spontaneously in the small intestine by the removal of IL-1Ra in mice.

## RESULTS

### Histological analysis

#### Crypt-villus axis height and villus width

**Figure 1:**
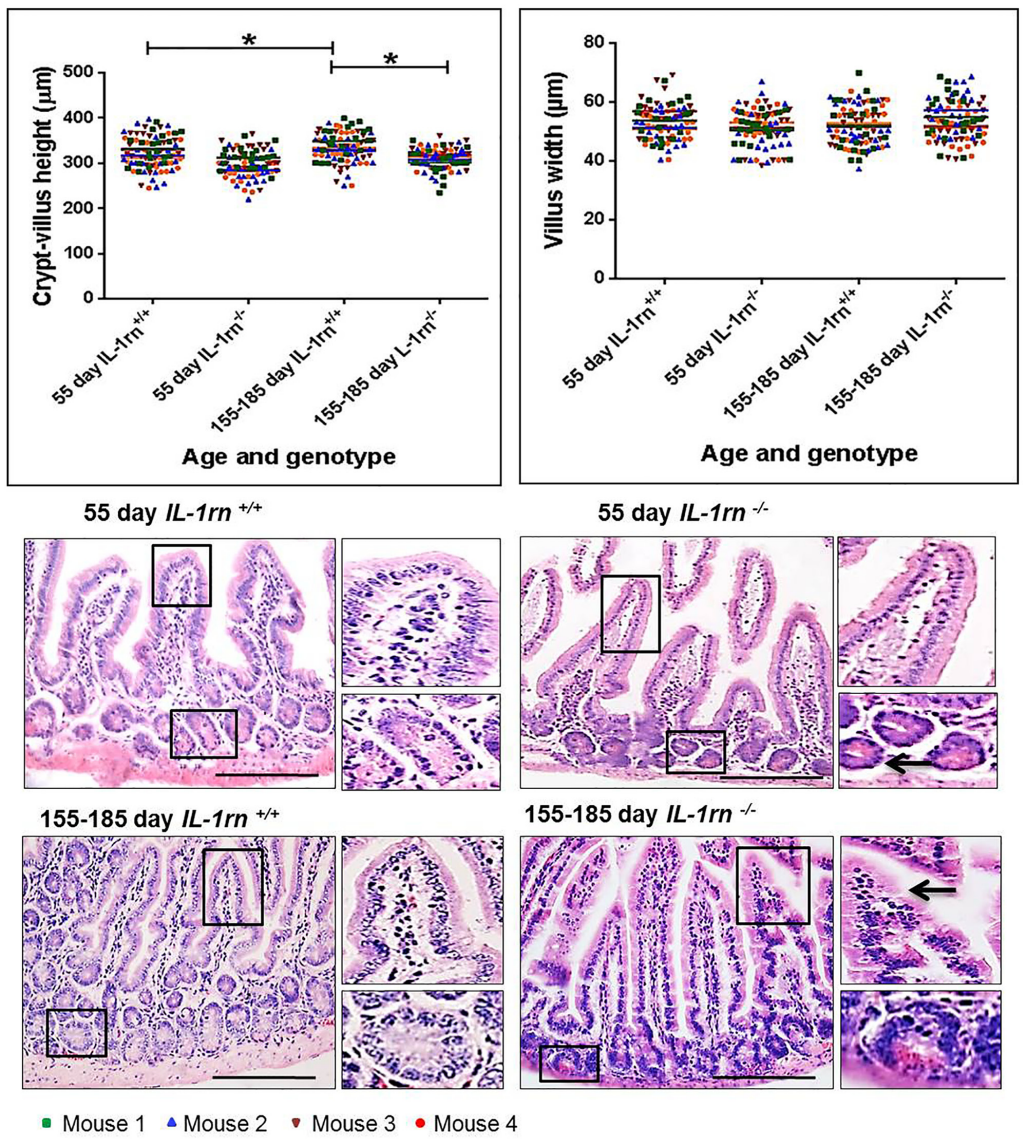
Histological analysis and morphology of the intact well-oriented crypt-villus axis heights and villus widths of Jejunum in the 55 day old IL-1rn^-/-^ mice and 155-185 day old IL-1rn^-/-^ mice compared to age-matched wild-type mice. Stained with H&E. Black arrows indicate moderate epithelial damage in 155-185 day old IL-1rn^-/-^ mice, enlarged space between the crypt base and the muscularis mucosa. ^*^P ≤ 0.05. Scale bar = 100 µm.

In the jejunum, there was a significant decrease in height of crypt-villus axis of the 155-185 day old *IL-1rn^-/-^* mice compared with age-matched WT mice (~7% decrease)(P≤0.05) (Figure 1). However, the crypt-villus height of the jejunum was significantly greater in the 155-185 day old compared to the 55-day old mice in WT groups (P≤0.05). The width of the villus at half crypt-villus axis was unchanged in all groups (Figure 1). Morphology of jejunal villi of 155-185 day old *IL-1rn^-/-^* mice revealed moderate epithelial damage with separation of the columnar epithelia from the lamina propria within the villi and the formation of large spaces between the crypt base and the muscularis mucosa in the 55-day old *IL-1rn^-/-^* mice (Figure 1).

**Figure 2:**
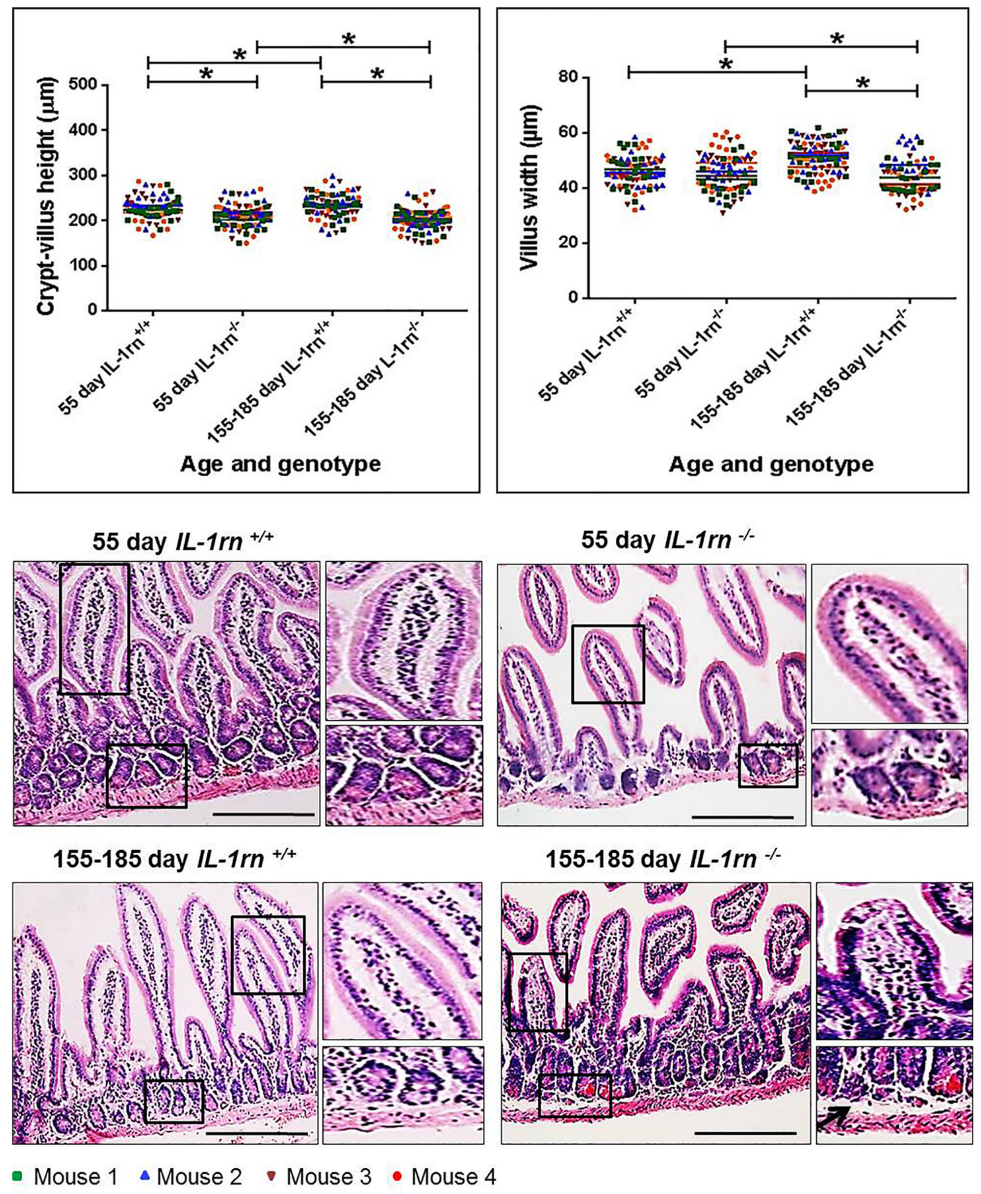
Histological analysis and morphology of the intact well-oriented crypt-villus axis heights and villus widths of Ileum in the 55 day old IL-1rn^-/-^ mice and 155-185 day old IL-1rn^-/-^ mice compared to age-matched wild-type mice. Stained with H&E. Black arrows indicate enlarged space between the crypt base and the muscularis mucosa. ^*^P ≤ 0.05. Scale bar = 100 µm.

In the ileum, there was a significant decrease in the crypt-villus axis height of both the 55-day old (~7% decrease) and 155-185 day old (~13% decrease) *IL-1rn^-/-^* mice compared with WT mice (P≤0.05) and a significant decreased in 155-185 day old *IL-1rn^-/-^* mice compared with 55-day old *IL-1rn^-/-^* mice (P≤0.05). In contrast, the crypt-villus axis height was significantly increased in 155-185 day old *IL-1rn^+/+^* mice compared with 55-day *IL-1rn^+/+^* mice (Figure 2). A significant decrease was seen in villus width in 155-185 day old *IL-1rn^-/-^* mice compared with 155-185 day old WT mice (~15% decrease) and 55-day *IL-1rn^-/-^* mice (P≤0.05). While villus width in the ileum was significantly increased in 155-185 day WT mice compared with 55-day old WT mice (P≤0.05) (Figure 2). Once again there was a separation of the columnar epithelium from the lamina propria within the villi and the formation of large spaces between the crypt base and the muscularis mucosa in the 155-185 day old *IL-1rn^-/-^* mice (Figure 2).

#### Number of goblet cells per crypt-villus axis

**Figure 3:**
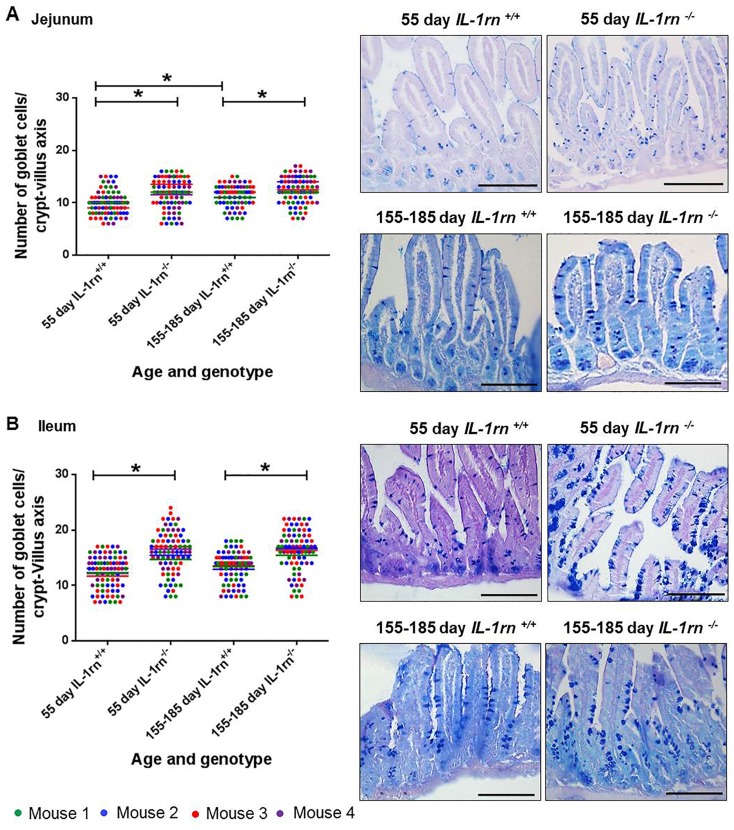
Histological analysis and morphology of goblet cells per intact well-oriented crypt-villus axis of the **(A)** Jejunum and **(B)** Ileum of 55 day old and 155-185 days old IL-1rn^-/-^ mice compared with age-matched WT mic, stained with AB-PAS. ^*^P ≤ 0.05. Scale bar = 100 µm.

In the jejunum and ileum, there was a significant increase in the number of goblet cells per crypt-villus axis in all *IL-1rn^-/-^* mice groups compared with WT mice (P≤0.05). Furthermore, in the jejunum, there was a significant increase in the number of goblet cells per crypt-villus axis in 155-185 day old WT compared to 55 day old WT mice (P≤0.05) (Figure 3A & 3B). Moderate and intense PAS (pink) staining was observed in 55 day old WT and *IL-1rn^-/-^* mice and intense alcian blue staining was observed in 155-185 day old WT *and IL-1rn^-/-^* mice. This indicated the presence of neutral mucins in younger mice, and acidic mucins in older mice in both the jejunum and the ileum (Figure 3A & 3B).

#### Assessment of pro-inflammatory cytokine expression and localization

**Figure 4:**
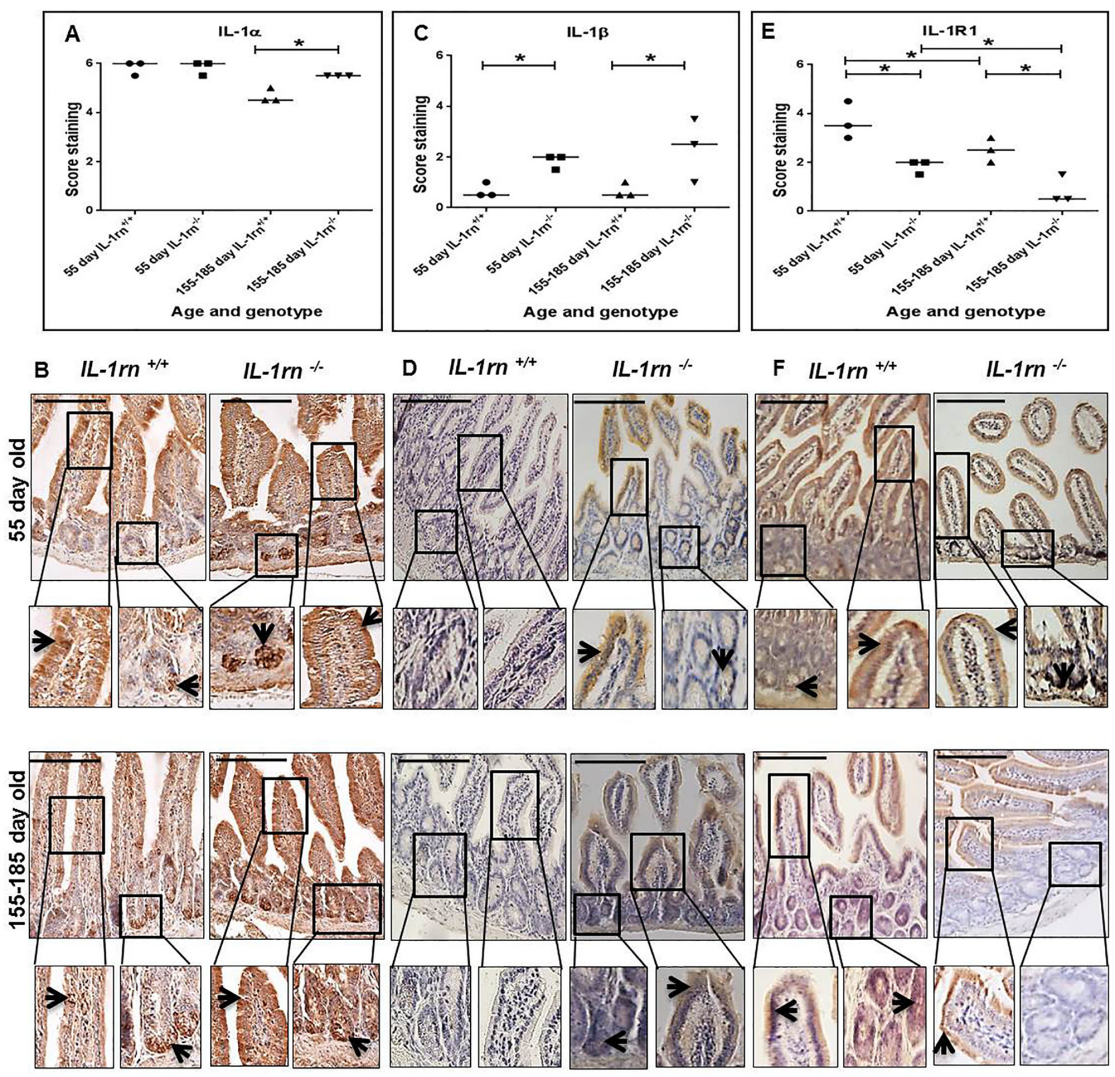
Immunohistochemistry staining of the expression and localization of pro-inflammatory cytokines: IL-1α **(A)** IL-1β **(C)** IL-1RI **(E)** show the immunopositive intensity quantification across the small intestinal architecture. **(B, D)** and **(F)** showing the immunopositive staining in the ileum of the 55 day old and 155-185 day old IL-1rn^-/-^ mice compared with WT mice. Cell nuclei were stained with haematoxylin (blue). Black arrows indicate immunopositivity. ^*^P ≤ 0.05. Scale bar = 100 µm.

Across the small intestine (jejunum and ileum), the expression of pro-inflammatory cytokine: IL-1α was highly expressed in both the villi and crypts. There was a significant increase in IL-1α immunopositivity in 155-185 day old *IL-1rn^-/-^* mice, compared with WT mice and 55 day *IL-1rn^-/-^* mice (P≤0.05) (Supplementary Figure 1A and Figure 4A & 4B). Whilst high levels of IL-1α were seen in all mice, only low levels of immunopositivity were seen in WT mice with a significant increase in IL-1β in the 55 day old and 155-185 day old *IL-1rn^-/-^* mice, compared with WT mice (P≤0.05) (Supplementary Figure 1B and Figure 4C & 4D). The expression of IL-1R1 was primarily located in villi (Supplementary Figure 1C and Figure 4E & 4F); however, there was a significant decrease in IL-1R1 expression in both the 55 day old and 155-185 day old *IL-1rn^-/-^* mice compared with WT mice (P≤0.05). IL-1R1 expression in 155-185 day old WT and 155-185 day old *IL-1rn^-/-^* mice were significantly lower than those seen in the 55 day old WT and 55 day old *IL-1rn^-/-^* mice, respectively (P≤0.05) (Supplementary Figure 1C and Figure 4E & 4F).

**Figure 5:**
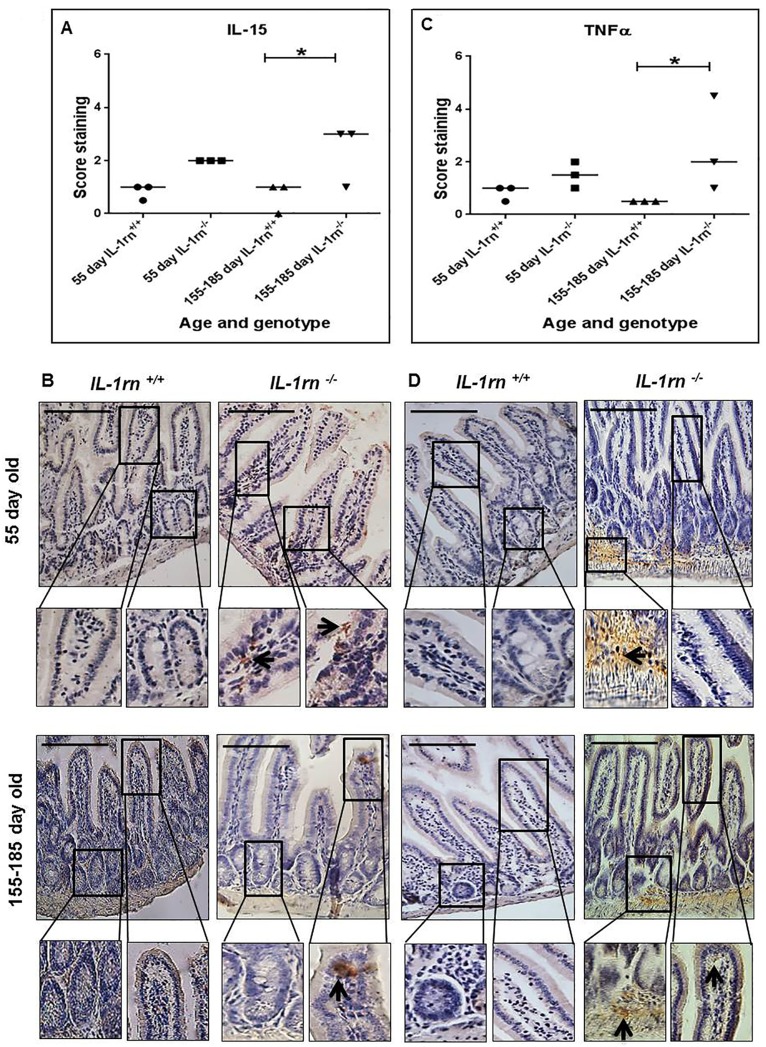
Immunohistochemistry staining of the expression and localization of pro-inflammatory cytokines: IL-15 **(A)** TNFα **(C)** show the immunopositive intensity quantification across the small intestinal architecture. **(B** and **D)** showing immunopositive staining in the ileum of the 55 day old and 155-185 day old IL-1rn^-/-^ mice compared with WT mice. Cell nuclei were stained with haematoxylin (blue). Black arrows indicate immunopositivity. ^*^P ≤ 0.05. Scale bar = 100 µm.

IL-15 expression was observed only at low levels in the 55 day old *IL-1rn^-/-^* mice, and localized in both the villi and crypts, but significantly increased in the 155-185 day old *IL-1rn^-/-^* mice, compared with WT mice (P≤0.05) (Supplementary Figure 2A and Figure 5A & 5B). TNFα expression was also observed at low levels in the 55 day old *IL-1rn^-/-^* mice. TNFα was localized in the villi of the jejunum and in the submucosa of the ileum, and was expressed at greater levels than in the WT mice. Whereas TNFα expression was significantly increased in the 155-185 day old *IL-1rn^-/-^* mice, where immunopositive cells were localized within the villi, crypts, and the submucosa of the jejunum and ileum, and was significantly greater than that seen in the WT mice (P≤0.05) (Supplementary Figure 2A and Figure 5C & 5D).

**Figure 6:**
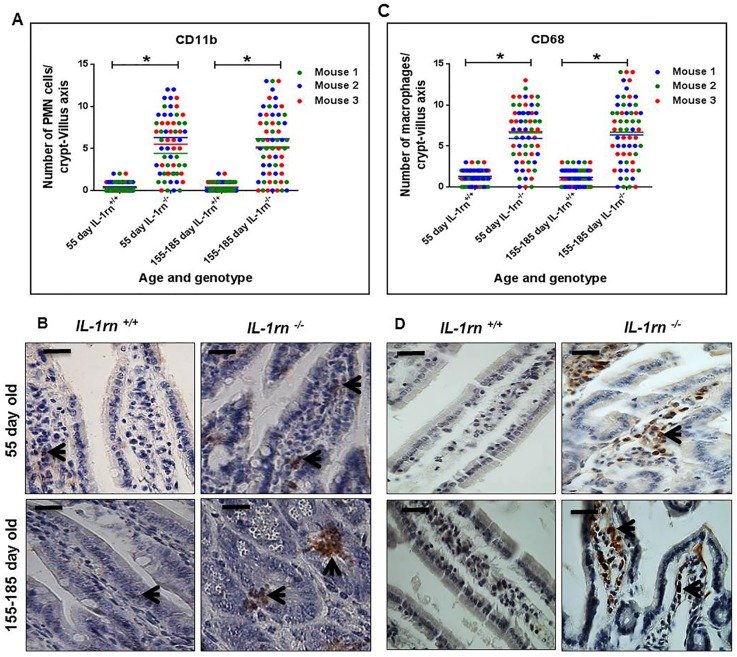
Immunohistochemistry staining of the infiltrated cells: Polymorphonuclear cells (PMNs) **(A** and **B)** and macrophage cells **(C** and **D)** into the lamina propria of the intact well oriented crypt-villus axis in the ileum of the 55 day old and 155-185 day old IL-1rn^-/-^ mice compared with WT mice. Cell nuclei were stained with haematoxylin (blue). Black arrows indicate immunopositivity. ^*^P ≤ 0.05. Scale bar = 25 µm.

The number of infiltrated polymorphonuclear (PMN) cells in the lamina propria were significantly increased in the 55 day old and 155-185 day old *IL-1rn^-/-^* mice compared with WT mice (P≤0.05) (Supplementary Figure 3A and Figure 6A & 6B). Crypt abscesses were observed in the ileum of 155-185 day old *IL-1rn^-/-^* mice. Here, PMN cells were seen to cluster together within the lamina propria of the villi of 55 day old *IL-1rn^-/-^* mice and in the crypts of the 155-185 day old *IL-1rn^-/-^* mice. The number of macrophages was significantly increased in 55 day old and 155-185 day old *IL-1rn^-/-^* mice compared with WT mice (P≤0.05). Similar distributions were seen throughout the lamina propria of the villi in the young and old *IL-1rn^-/-^* mice in the jejunum and ileum (Supplementary Figure 3B and Figure 6C & 6D).

#### Assessment of degrading enzyme expression and localization

**Figure 7:**
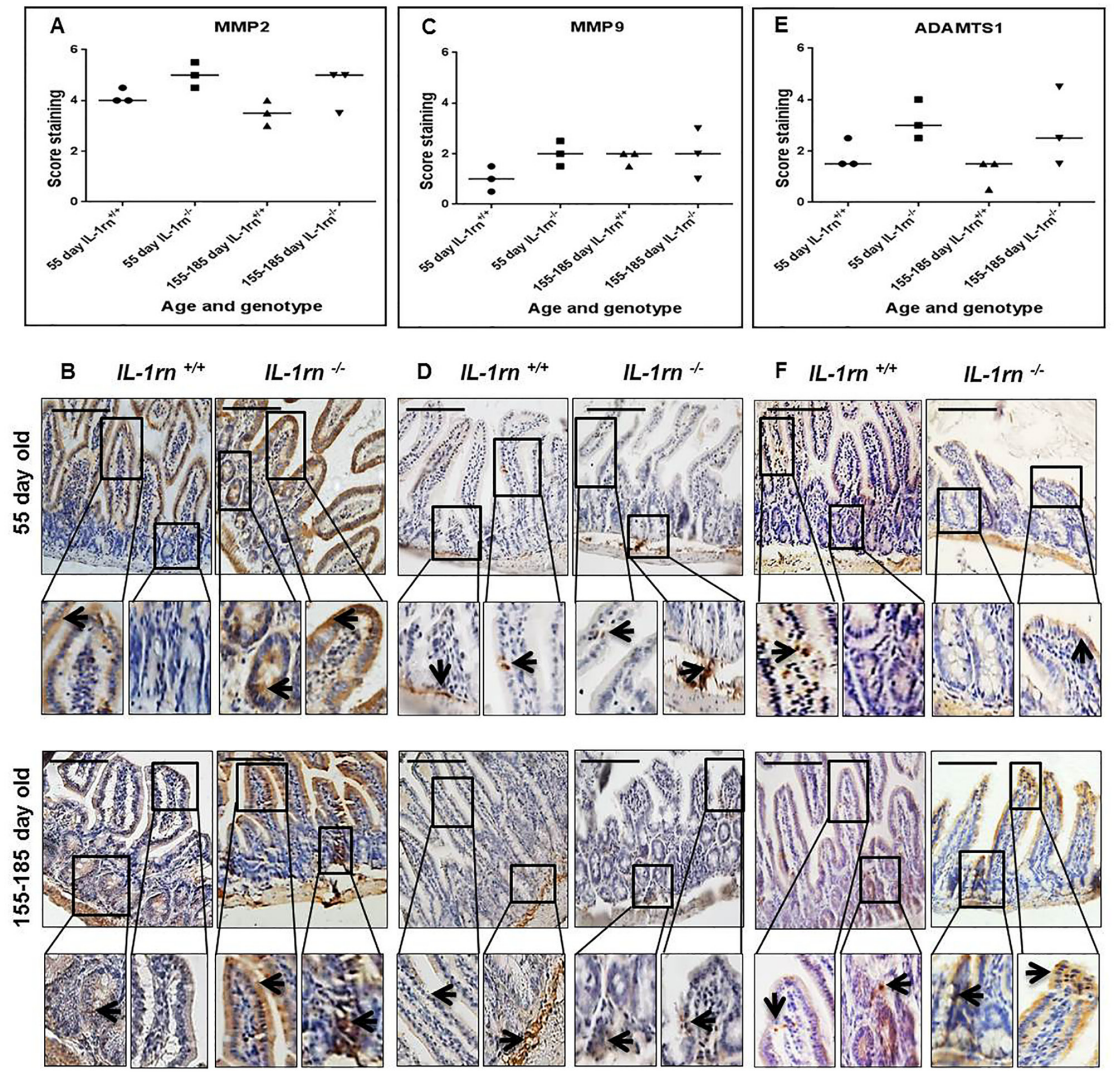
Immunohistochemistry staining of the expression and localization of degrading enzymes: MMP2 **(A)** MMP9 **(C)** ADAMTS1 **(E)** show the immunopositive intensity quantification across the small intestinal architecture. **(B, D** and **F)** showing the immunopositive staining in the ileum of the 55-day old and 155-185 day old IL-1rn^-/-^ mice compared with WT mice. Cell nuclei were stained with haematoxylin (blue). Black arrows indicate immunopositivity. Scale bar = 100 µm.

Although MMP2 expression was higher in both the 55 day old and 155-185 day old *IL-1rn^-/-^* mice than WT mice, this failed to reach significance. In 55 day old and 155-185 day old *IL-1rn^-/-^* mice, MMP2 was highly expressed in the villi, crypts and muscularis mucosa (Supplementary Figure 4A and Figure 7A and 7B).

MMP9 expression was limited in all groups; its expression was higher in the submucosa of the ileum than the jejunum in the 55 day old WT, *IL-1rn^-/-^* mice and in 155-185 day old WT mice (Supplementary Figure 4B and Figure 7C and 7D). Although ADAMTS1 was highly expressed in the jejunum and ileum of the *IL-1rn^-/-^* mice, there were no significant differences between age and genotypes (Supplementary Figure 4C and Figure 7E and 7F).

#### Assessment of enterocyte polarity and cell-cell adherence

**Figure 8:**
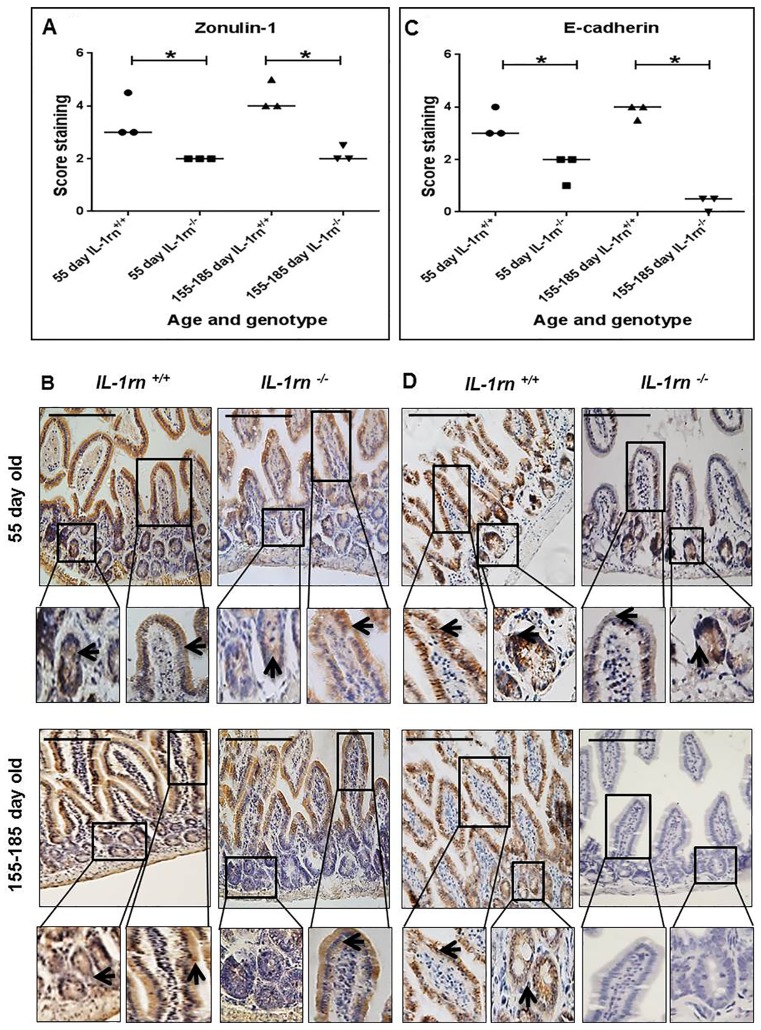
Immunohistochemistry staining of the expression and localization of junctional proteins: ZO-1 **(A)** E-cadherin **(C)** showing the immunopositive intensity quantification across the small intestinal architecture. **(B** and **D)** show the immunopositive staining in the ileum of the 55 day old and 155-185 day old IL-1rn^-/-^ mice compared with WT mice. Cell nuclei were stained with haematoxylin (blue). Black arrow sindicate immunopositivity. ^*^P ≤ 0.05. Scale bar = 100 µm.

ZO-1 and E-cadherin were expressed from the surface of the villi down to crypts in WT mice. The level of expression of both ZO-1 and E-cadherin was significantly decreased in both the 55 day old and 155-185 day old *IL-1rn^-/-^* mice compared to age-matched WT controls (P≤0.05) (Supplementary Figure 5A & 5B and Figure 8A, 8B, 8C, 8D). In the jejunum, ZO-1 expression was localized on villi tips only in the 155-185 day old *IL-1rn^-/-^* mice, which was significantly less than that seen in younger 55 day old of *IL-1rn^-/-^* and WT mice (Supplementary Figure 5A). In contrast, ZO-1 was uniformly expressed at the cell surface of the entire villi in the ileum and was comparable to that seen in WT mice (Figure 8A and 8B). E-cadherin immunopositivity was lost in the jejunum and ileum of 155-185 day old of *IL-1rn^-/-^* mice compared to WT mice (Supplementary Figure 5B and Figure 8C & 8D).

#### Assessment of digestive enzyme expression and localization

**Figure 9:**
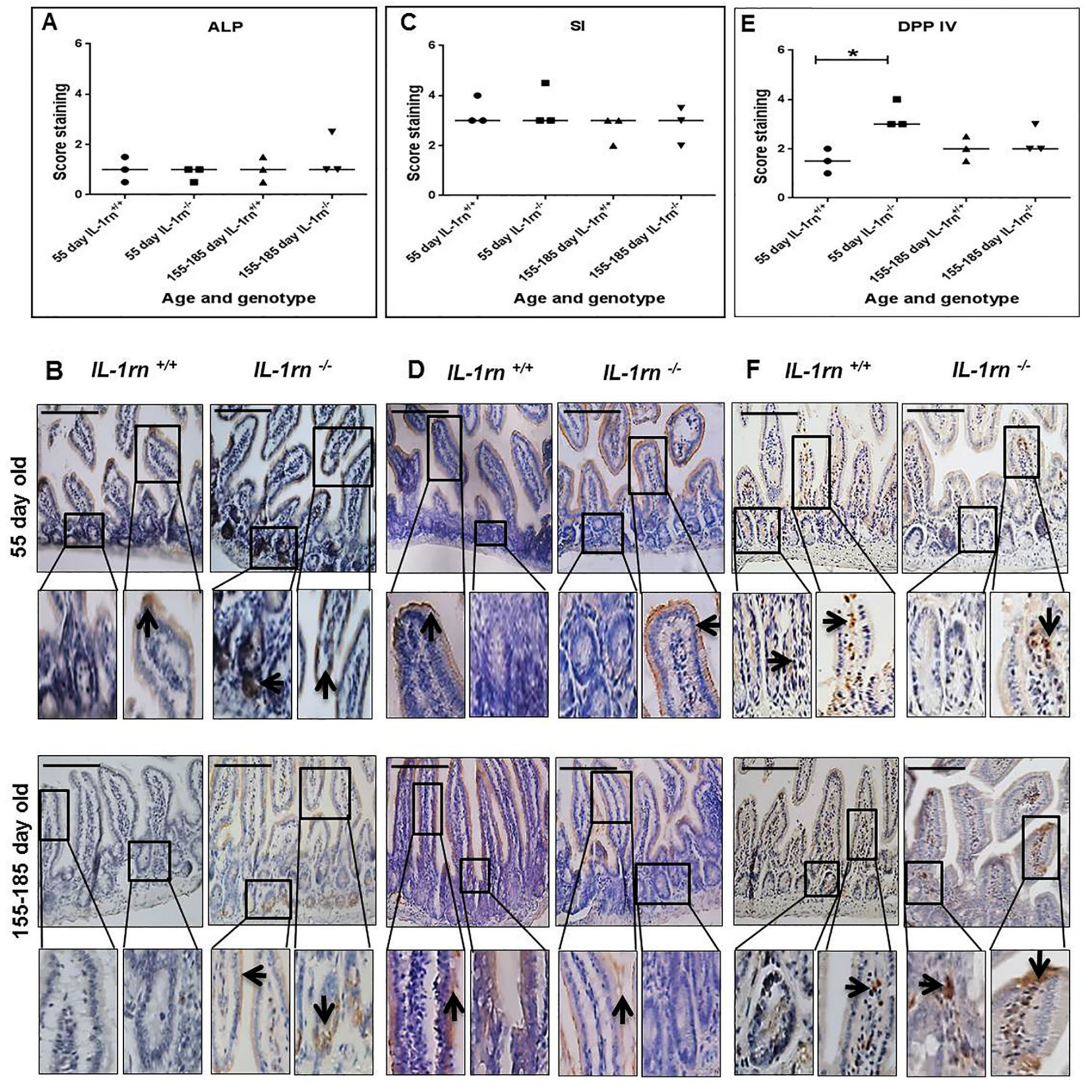
Immunohistochemistry staining of the expression and localization of the digestive enzymes: ALP **(A)** SI **(C)** and DPP IV **(E)** show the immunopositive intensity quantification across the small intestinal architecture. **(B, D** and **F)** showing the immunopositive staining in the ileum of the 55 day old and 155-185 day old IL-1rn^-/-^ mice compared with WT mice. Cell nuclei were stained with haematoxylin (blue). Black arrows indicate immunopositivity. ^*^P ≤ 0.05. Scale bar = 100 µm.

ALP expression was weak in all mice; however, specific ALP immunopositivity was seen on the brush border of the enterocytes and in the lamina propria cells (Supplementary Figure 6A and Figure 9A & 9B).

Sucrase-isomaltase expression was localized to the apical epithelial surface and was highly expressed in all mice (Supplementary Figure 6B and Figure 9C & 9D). DPP IV expression was increased in 55-day old IL-1rn-/- mice compared with WT mice (P≤0.05), this expression was localized on the enterocyte surface and in the lamina propria cells (Supplementary Figure 6C and Figure 9E & 9F).

## DISCUSSION

The balance between IL-1 and IL-1Ra as an endogenous inhibitor plays an essential role in stimulation and regulation of inflammation in IBD [1], however, whether IL-1 is an important causative factor in IBD or purely a result of inflammation is not currently understood. Thus, this study investigated whether changes seen during IBD could be induced spontaneously by the removal of IL-1Ra in mice that lack a functional *IL-1rn* gene. In *IL-1rn^-/-^* mice, the villi were shown to be shorter in the jejunum, while shorter and thinner in the ileum, within the ileum of older mice a 13% decrease in height and 15% decrease in width was observed. These large changes would result in a substantial decrease in digestive and absorptive area, and thus be expected to lead to defiency in nutrient uptake. This blunting of the villi is a common microscopic feature of IBD and has been observed in patients with Crohn's disease in both the jejunum and the ileum [12]. In contrast, in WT mice the villus height and width were shown to be comparable to those of wild-type C57BL/6J mice of a similar age and region of the small intestine [13]. As seen in the WT mice, there was a decrease in height and width along a proximal-distal gradient of the small intestine. Similarly to these findings, in a wild-type C57BL/6J mice model a comparison of old and young mice, showed an increase in villi height with age, whilst the villi width was unchanged [13].

Physiologically intestinal goblet cells secrete large amounts of mucus, which coat and cover the intestinal villi. This mucus plays an important role in the maintenance of the intestinal mucosal barrier and creates a milieu in which digestion and absorption take place [14]. Here, a substantial increase in the number of goblet cells per villus in both jejunum and ileum of *IL-1rn^-/-^* mice compared with WT mice was observed which reached 22-29% more goblet cells in the ileum. This large increase in goblet cells which accompanies the inflammatory process is believed to be caused by increased expression of the transcription factors: Hath1 (a basic helix-loop-helix (bHLH) transcription factor) and Klupper like factor 4 (KLF4), which are essential for goblet cell differentiation [15]. This inflammatory process is believed to drive the increase in goblet cell differentiation, which is observed in Crohn's disease patients [15]. However, it is important to note that this increase in goblet cells is not found in all Crohn's disease patients, or throughout the whole gastrointestinal tract [16].

Maintenance of intestinal homeostasis is regulated by epithelial barrier integrity [17]. Diffusion of the luminal materials into lamina propria promotes a local inflammatory response causing release of proinflammatory cytokines, release of MMPs, and epithelial degradation and inflammation [18]. Expression of pro-inflammatory cytokines: IL-1α, IL-1β, TNFα, and IL-15 were increased in *IL-1rn^-/-^* mice, and these results are in agreement with those obtained from patients with Crohn's disease [19]. IL-1α and IL-1β are also expressed in human intestinal epithelial cells [20]. IL-1α is released when these human intestinal cells are damaged or destroyed, and IL-1α was commonly detected in the epithelium of IBD, released from necrotic intestinal epithelial cells and plays a crucial role in intestinal inflammation by inducing human intestinal fibroblasts to produce IL-6 and IL-8 [21]. The high concentrations of IL-1β in the intestine of patients with Crohn's disease are mainly attributed to local mononuclear cell infiltration [22], which were also observed in this model. Increased production of IL-1β and TNFα in inflamed Crohn's disease mucosa induces the synthesis of IL-8, which is an effective neutrophil chemoattractant [23]. These findings agree with previous work, which showed increased numbers of TNFα positive cells in the lamina propria and submucosa of patients with Crohn's disease. Furthermore, TNFα produced by subepithelial macrophages has been shown to contribute to decreased epithelial integrity [24]. TNFα acts as an important mediator of inflammation through MAPK and NF-κB activation, increasing cell proliferation and altering epithelial barrier permeability [25]. The production of TNFα is also high in cultured mucosal mononuclear cells from Crohn's disease patients [26]. However, in contrast a previous study observed no differences in TNFα mRNA expression between control and IBD mucosal biopsies [27].

IL-1RI is expressed on many cell types including T cells, fibroblasts, and intestinal epithelial cells. Despite the increased expression of IL-1β in the *IL-1rn^-/-^* mice, the expression of IL-1R1 was reduced; this could suggest a negative feedback loop decreasing expression of IL-1RI in the presence of uncontrolled IL-1 production. This agrees with a previous study, which showed IL-1β and TNFα treatment downregulated the expression of mRNA IL-1RI in rat intestinal epithelial cells (IEC-6) [28]. Similarly, IL-1β treatment was also shown to downregulate IL-1RI protein in retinal endothelial cells (TR-iBRB2) [29]. This is contrary to previous study, in which an increase in IL-1β induced chronic intestinal inflammation in mice and an increase in IL-17A-secreting innate lymphoid cells which express high levels of IL-1R1 [30]. These differences may be a result of the different levels of inflammation seen in these models. Here, increased expression of IL-15 was seen in *IL-1rn^-/-^* mice, this is in agreement with Liu *et al* (2000), who showed increased expression of IL-15 by macrophages in the inflamed ileum of patients with Crohn's disease and that IL-15 increased local T cell activation and induced proinflammatory cytokine production by T cells and macrophages. The presence of polymorphonuclear cells and mononuclear cells play an important role in the augmentation of inflammation and tissue damage in IBD [31]. Increased infiltration of these CD11b and CD68 positive cells into the lamina propria were observed in *IL-1rn^-/-^* mice compared with WT mice. These findings are in agreement with previous findings which showed increased numbers of these cells in the inflamed mucosa of IBD patients [32]. Infiltration of immune cells such as macrophages and lymphocytes in the lamina propria is an important aspect of IBD, especially Crohn's disease, even in the lack of noticeable morphological, clinical, and endoscopic indication of inflammation. This is thought to be due to an increased demand of macrophages within the inflamed intestine [33]. Functional assessment of this increased inflammatory response such as calprotectin measurement in stool samples would have been a useful assessment, however unfortunately these samples were not available in this study.

MMPs and ADAMTS1 expression were not significantly altered in *IL-1rn^-/-^* mice. MMPs have been demonstrated to be induced in several pathological conditions of IBD and play a key role in regulating the pathophysiology of IBD [34]. The main action of MMPs is to degrade extracellular matrix proteins. Active MMP2 and MMP9 play an important role in cell migration and cytokine stimulation. Indeed neutrophil infiltration has been shown to be induced by upregulation of MMP9 in murine inflamed intestine [35]. Furthermore, stromal cells play an important role in intestinal inflammation and the pathogenesis of IBD. These cells secrete MMP2 and MMP9 [36]; and MMP9 is produced by human colonic epithelium during IBD [37]. In addition, infiltrating macrophages and neutrophils are the main source of MMP9 in human IBD [38]. Overexpression of MMP9 has been shown to cause a reduction in the differentiation of progenitor cells to goblet cells and consequently decreased MUC2 expression, which leads to decreases in the protective mucin barrier in colonic epithelium [35].

The maintenance of epithelial barrier function and control of paracellular permeability are regulated by tight junction proteins [39]. Epithelial disruption and decrease in tight junctions that lead to increased intestinal permeability are the common features of Crohn's disease [40]. Decreased expression of ZO-1 and loss of E-cadherin expression were observed in *IL-1rn^-/-^* mice, suggesting a dysfunctional and leaky epithelial barrier, and separation of the mucosa and lamina propria. Inflammatory cytokines are known to affect epithelial junctional complexes, causing barrier dysfunction and increased epithelial permeability [39]. A previous study showed that a tight junction disorder has been found in the epithelial cells of the terminal ileum from patients with Crohn's disease [41]. In addition, it has been shown that IL-1β and TNFα induced defects in intestinal epithelial tight junctions resulting in increased intestinal permeability [42].

ALP plays an essential role in maintaining small intestinal homeostasis and has been shown to prevent the activation of NF-kB, thus inhibiting release of pro-inflammatory cytokines. Decreased activity of ALP could increase intestinal inflammation [43], and intestinal epithelial dysfunction in IBD patients has been attributed to lower intestinal expression and activity of ALP [44]. Surprisingly, the expression of digestive enzymes were not altered in IL-1rn^-/-^ mice compared with WT mice. This demonstrated the resilience of the intestine to maintain function even during severe inflammation. SI expression was unchanged, which is contrary to decreased gene expression of SI in villus enterocytes seen in the ileum of patients with Crohn's disease [45]. DPP IV expression, however, was significantly increased in the young *IL-1rn^-/-^* mice, but not the older *IL-1rn^-/-^* mice compared to WT mice. A similar loss of DPPIV activity has been seen in the serum of patients with IBD [46].

## MATERIALS AND METHODS

### *IL-1rn^-/-^* BALB/c mice

*IL-1rn^tm1Nick^* deficient mice (*IL-1rn^-/-^*) have been described previously [47]. All mice were housed behind positive pressure barriers and were reared under UK Home Office licenses. All materials were supplied sterile and certified pathogen free. This work was approved by the University of Sheffield ethical review panel. Formalin-fixed mice were a kind gift from Dr. Martin Nicklin, University of Sheffield. To ensure that experimental procedures aligned with the 3Rs principle, initial work was completed on n=4 and statistical analysis performed, as statistically significant differences were seen in n=4 it was not deemed ethically appropriate nor necessary to increase the number of animals utilised.

### Tissue preparation and histological assessment

The entire jejunum and ileum portions of small intestine were dissected from BALB/c *IL-1rn^-/-^* knockout mice aged 50-55 days (n=4) and 155-185 day old (n=4), together with age-matched BALB/c wild-type (WT) controls (n=4 at each age). Following dissection, tissues were rinsed in PBS and fixed in 10% v/v formalin for 24 h and then transferred to 70% v/v ethanol. Tissues were processed and embedded in paraffin wax. Five-micron sections were cut and mounted onto positively charged slides (Leica Microsystem Milton Keynes, UK). Sections were deparaffinised in Sub-X and rehydrated in industrial methylated spirits (IMS) prior to rehydration in distilled water. Sections were then stained with either: Haematoxylin and Eosin; Mayer's Haematoxylin (Leica Microsystem, Milton Keynes, UK) for 2 min rinsed in water for 5 min and immersed in Eosin (Leica Microsystem, Milton Keynes, UK) for 1 min; or Alcian Blue/Periodic acid Schiff's (PAS): 1% w/v Alcian Blue (PH 2.5) (Sigma-Aldrich, Poole, UK) in 3% (v/v) acetic acid (Sigma-Aldrich, Poole, UK) for 30 min and immersed in 0.5% (w/v) Periodic acid for 10 min and rinsed three times in deionised water. Slides were then immersed in Schiff reagent (Merck KGaA, Germany) for 10 min, and then rinsed three times with deionised water. Following staining, sections were dehydrated in IMS, cleared with Sub-X and mounted in Pertex (Leica Microsystem, Milton Keynes, UK). The slides were examined with an Olympus BX 51 microscope and images captured by the digital camera and Capture Pro OEM V8.0 software (Media Cybernetics, Buckinghamshire, UK).

The tissue morphology was assessed using the Capture Pro OEM V8.0 software measurement tools as follows:

### Crypt-villus height and villus width

Twenty well oriented crypt-villus axes height and villus width at half-axis height in longitudinal tissue sections were measured in the jejunum and ileum of all mice, from each age range and genotype.

### Goblet cells per crypt-villus axis

The numbers of goblet cells within twenty well oriented crypt-villus axes were counted in the jejunum and ileum of all mice from each age range and genotype.

### Immunohistochemical assessment

#### Pro-inflammatory cytokine expression and immune cell infiltration

The expression of pro-inflammatory cytokines IL-1α, IL-1β, and their receptor: IL-1RI; IL-15, and TNFα were investigated by immunohistochemistry. In addition, the number of immune cells which infiltrated into lamina propria of crypt-villus axis was determined using immunohistochemistry of polymorphonuclear cell marker: CD11b and macrophage marker CD68, in the jejunum and ileum of three randomly selected mice, from each age range and genotype.

#### Matrix-degrading enzyme expression

The expression of matrix-degrading enzymes: MMP2, MMP9, and ADAMTS1 were assessed in the jejunum and ileum of three randomly selected mice, from each age range and genotype using immunohistochemistry.

#### Polarity of enterocytes and digestive enzyme expression

The expression of tight junction proteins Zonulin 1 (ZO-1) and adherent junction protein E-cadherin, were assessed alongside digestive enzymes: alkaline phosphatase (ALP), sucrase-isomaltase (SI), and dipeptidyl peptidase IV (DPP IV) in the jejunum and ileum of three randomly selected mice, from each age range and genotype.

Immunohistochemistry was performed as described previously [48]. Briefly, 5 µm sections were de-waxed, rehydrated, and endogenous peroxidase blocked using hydrogen peroxide (Sigma-Aldrich, Poole UK). After washing in Tris-buffered saline (TBS) (20mM Tris, 150mM sodium chloride, pH 7.5), tissue sections were subjected to antigen retrieval sections were subjected to an appropriate antigen retrieval method, specific to the antibodies investigated (Table 1). Following this sections were washed in TBS. Then nonspecific binding sites were blocked at room temperature for 90 min with 25% (w/v) serum (Abcam, Cambridge, UK) in 1% (w/v) bovine serum albumin in TBS. Sections were incubated overnight at 4°C with appropriate primary antibody (Table 1).

**Table 1:** Target antibodies used for IHC, their optimal concentration and antigen retrieval methods

Antibody	Clonality	Dilution	Antigen retrieval	Supplier	Catalogue No.
IL-1α	Rabbit polyclonal	1:100	Heat	Abcam	ab7632
IL-1β	Rabbit polyclonal	1:100	Heat	Abcam	ab9722
IL-1R1	Rabbit polyclonal	1:100	Enzyme	Abcam	ab106278
IL-15	Rabbit polyclonal	1:50	Enzyme	Abcam	ab7213
TNFα	Rabbit polyclonal	1:50	Enzyme	Abcam	ab6671
CD11b	Goat polyclonal	1:600	None	Abcam	ab62817
CD68	Mouse monoclonal	1:200	Enzyme	Abcam	ab955
MMP2	Rabbit polyclonal	1:800	Enzyme	Abcam	ab37150
MMP9	Rabbit polyclonal	1:25	Heat	Abcam	ab38898
ADAMTS1	Rabbit polyclonal	1:200	Enzyme	Abcam	ab39194
ZO-1	Rabbit polyclonal	1:50	Enzyme	Abcam	ab217334
E-cadherin	Mouse monoclonal	1:200	Heat	Abcam	ab76055
ALP	Rabbit monoclonal	1:200	Heat	Abcam	ab108337
SI	Mouse monoclonal	1:50	Heat	Santa Cruz	sc-393470
DPP I	Mouse monoclonal	1:50	Enzyme	Abcam	ab119346

For each immunohistochemistry method, a negative control was performed, replacing the primary antibody with either rabbit or mouse IgG (Abcam, Cambridge, UK) as appropriate, at a concentration equal to that of the primary antibody. Sections were washed in TBS and then incubated in 1:500 dilution of an appropriate biotinylated secondary antibody for 30 min at room temperature. Binding of the secondary antibody was visualised after exposure to horseradish peroxidase (HRP) streptavidin-biotin complex (Vector Laboratories, Peterborough, UK) for 30 min. Sections were washed in TBS, and treated with 0.08% (v/v) hydrogen peroxide in 0.65mg/ml 3, 3′-diaminobenzidine tetrahydrochloride (Sigma-Aldrich, Poole, UK) in TBS for 20 min. Sections were counterstained with Mayer’s haematoxylin, dehydrated, cleared and mounted in Pertex. Immunohistochemical staining were examined with an Olympus BX51 microscope and images captured by digital camera and Capture Pro OEM v8.0 software (Media Cybernetics, Buckinghamshire, UK). Immunopositive intensity across the small intestine architecture was independently semi-quantified by two assessors (CLM and NJM), blinded to animal genotype and age. A scale of 0 to 6 was utilised where 0 was no immunopositivity and 6 signifies maximum immunopositivity. The number of immunopositive (CD11b and CD68) immune cells were counted within twenty well oriented crypt-villus axes of each of three randomly selected mice, from each age range and genotype.

### Statistical analysis

Data was plotted using GraphPad Prism V6.0. Crypt-villus axes height and villus width were assessed for normality using Stats Direct program and found to be non-parametric and therefore statistical comparisons were performed by Kruskal-Wallis with a pairwise comparison (Conover-Inman) between ages and genotypes. Statistical significance was set at P ≤ 0.05, for statistical analysis the mean villi height, width and goblet cells per villi per mouse were utilised for statistical analysis. All replicates are shown with the median values for each mouse to demonstrate clearly the spread of replicates.

## CONCLUSIONS

IL-1Ra deficient mice *(IL-1rn^-/-^*) induced spontaneous intestinal inflammation which provides an effective approach to display features associated with IBD. Old *IL-1rn^-/-^* mice demonstrated a higher inflammatory response than young *IL-1rn^-/-^* mice. This model delivers evidence for a role of the imbalance between IL-1 and IL-1Ra in the pathogenesis of IBD and could provide a useful model for testing new therapies.

## SUPPLEMENTARY MATERIALS FIGURES



## Abbreviations

IBD: Inflammatory bowel disease
IL-1: Interleukin 1
IL-1Ra: Interleukin 1 receptor antagonist
IL-1R1: Interleukin 1 receptor 1
WT: Wild type
MMPs: Matrix-degrading enzymes
ADAMTS1: A disintegrin and metalloproteinase with thrombospondin motifs
CD: Crohn’s disease
UC: Ulcerative colitis
PMNs: Polymorphonuclear cells
AB: Alcian blue
PAS: Periodic acid schiff
TNFα: Tumor necrosis factor
ZO-1: Zonulin 1
ALP: Alkaline phosphatase
SI: Sucrase-isomaltase
DPP IV: Dipeptidyl peptidase 4
