# Supplementary material for: Interleukin 1 is a key driver of inflammatory bowel disease-demonstration in a murine IL-1Ra knockout model
Source: Oncotarget. 2019 May 28;10(37):3559–75. doi: 10.18632/oncotarget.26894 (PMC6544399; doi:10.18632/oncotarget.26894)
Supplement: Supplementary file 1 [file oncotarget-10-3559-s001.pdf]

Interleukin 1 is a key driver of inflammatory bowel disease-demonstration in a murine IL-1Ra knockout model

SUPPLEMENTARY MATERIALS

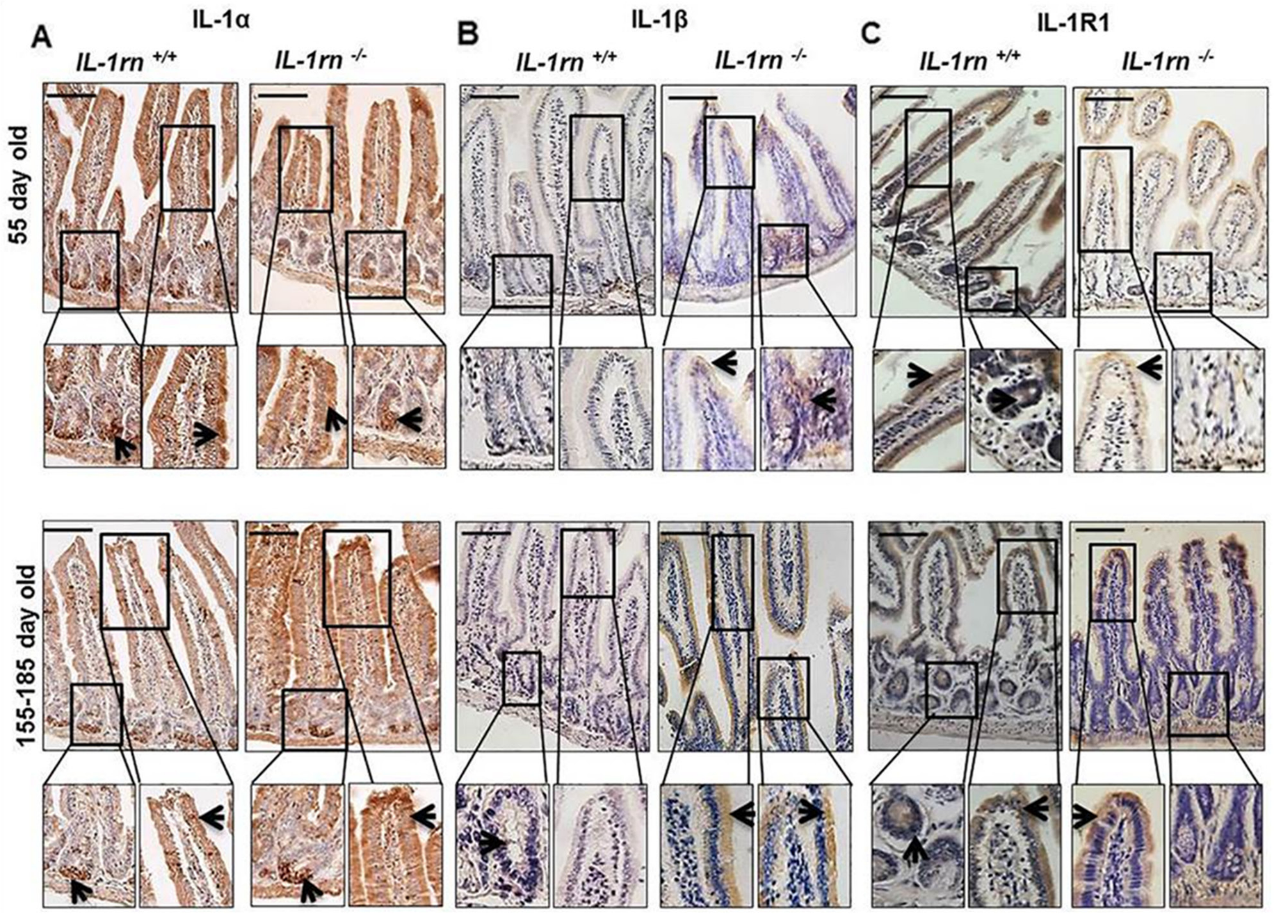

Supplementary Figure 1: Immunohistochemistry staining of the expression and localisation of pro-inflammatory cytokines. **(A)** IL-1α; **(B)** IL-1β; **(C)** IL-1RI in the jejunum of the 55-day old and 155-185 day old IL-$1rn^{-/-}$ mice compared with IL-$1rn^{+/+}$ mice. Cell nuclei were stained with haematoxylin (blue). Black arrows indicate immunopositivity. Scale bar = 100µm.

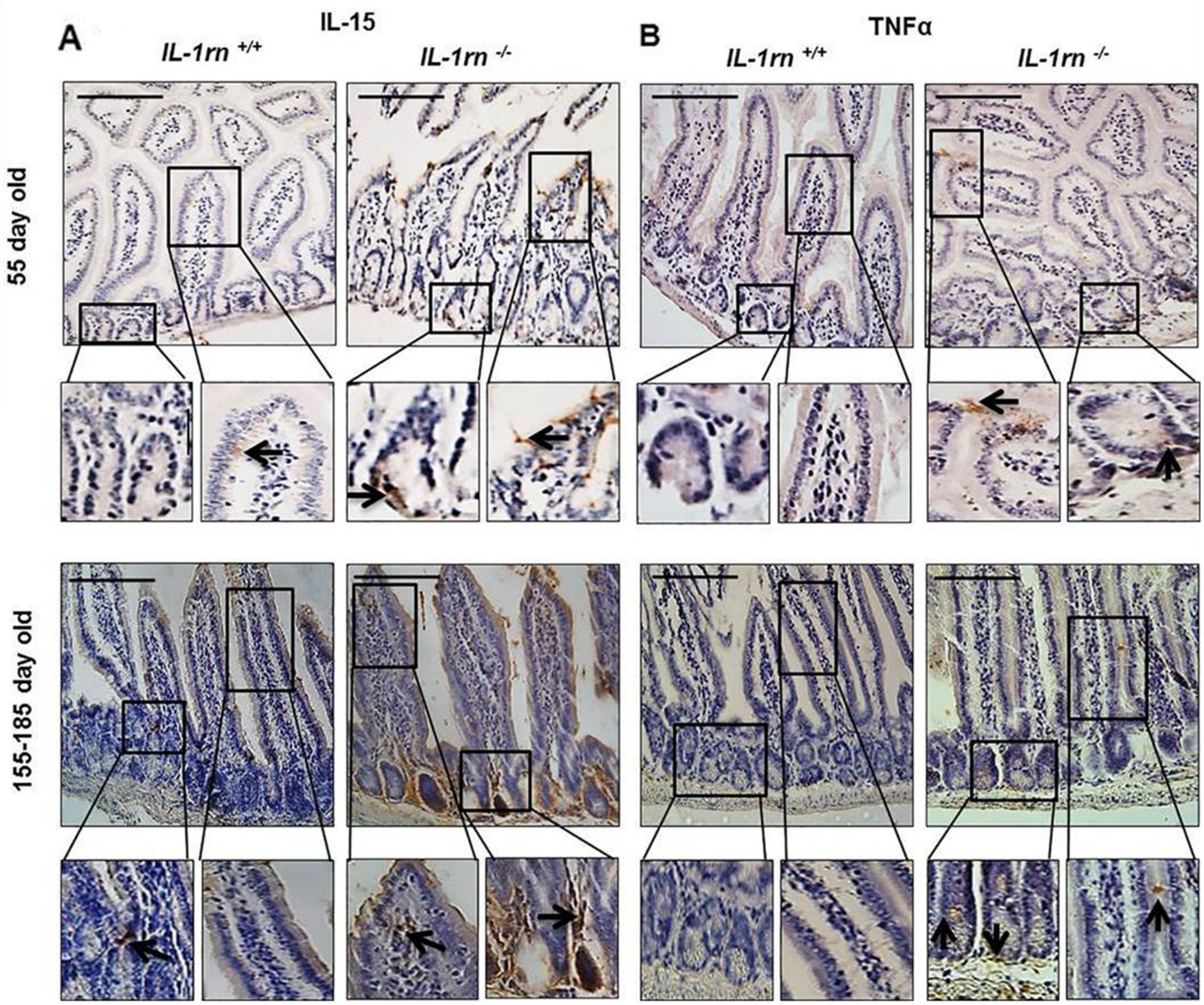

Supplementary Figure 2: Immunohistochemistry staining of the expression and localisation of pro-inflammatory cytokines. (A) IL-15; **(B)** TNFα in the jejunum of the 55-day old and 155-185 day old IL-1rn$^{-/-}$ mice compared with IL-1rn$^{+/+}$ mice. Cell nuclei were stained with haematoxylin (blue). Black arrows indicate immunopositivity. Scale bar = 100µm.

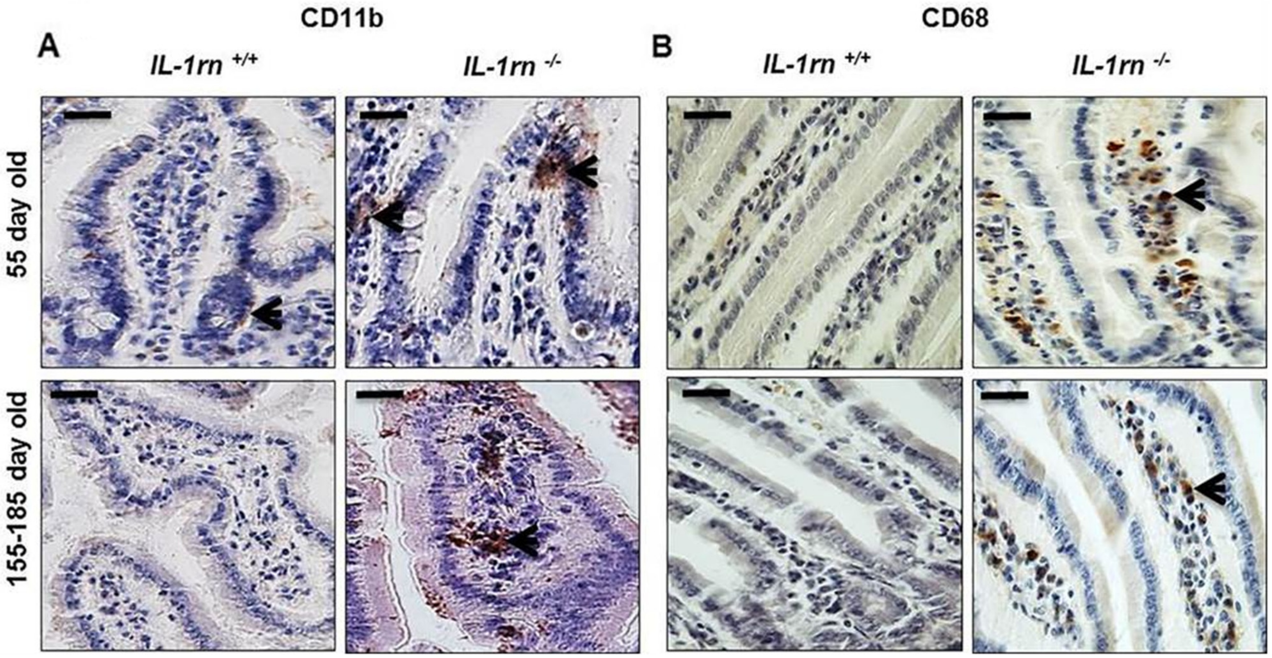

Supplementary Figure 3: Immunohistochemistry staining of infiltrating cells. **(A)** polymorphonuclear cells (PMNs) indicated by CD11b staining and **(B)** Macrophage cells (CD68) in the jejunum of the 55-day old and 155-185 day old IL-1rn$^{-/-}$ mice compared with IL-1rn$^{+/+}$ mice. Cell nuclei were stained with haematoxylin (blue). Black arrows indicate immunopositivity. Scale bar = 25μm.

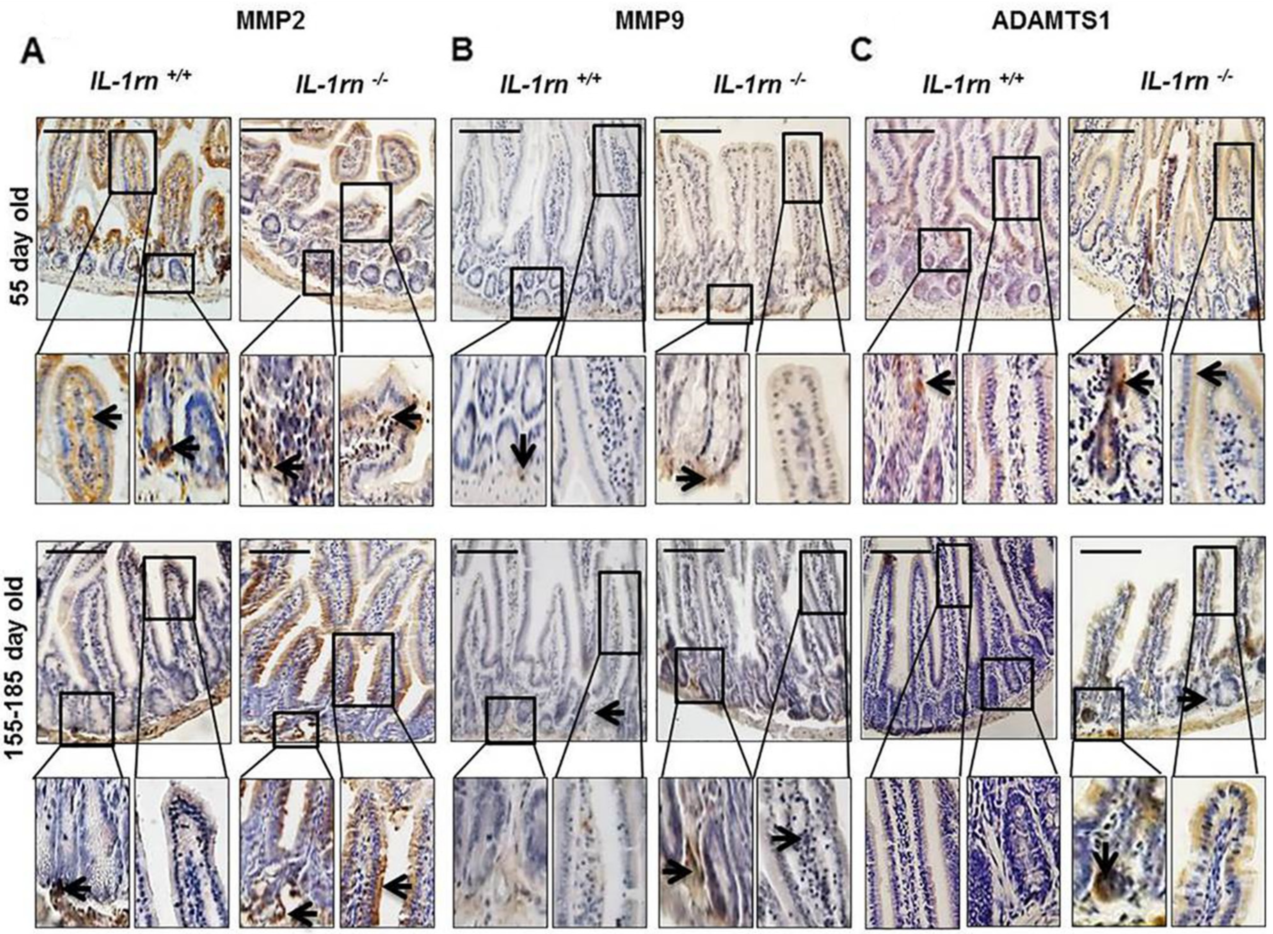

Supplementary Figure 4: Immunohistochemistry staining of the expression and localisation of degrading enzymes. (A) MMP2; **(B)** MMP9; **(C)** ADAMTS1 in the jejunum of the 55-day old and 155-185 day old IL-1rn$^{-/-}$ mice compared with IL-1rn$^{+/+}$ mice. Cell nuclei were stained with haematoxylin (blue). Black arrows indicate immunopositivity. Scale bar = 100µm.

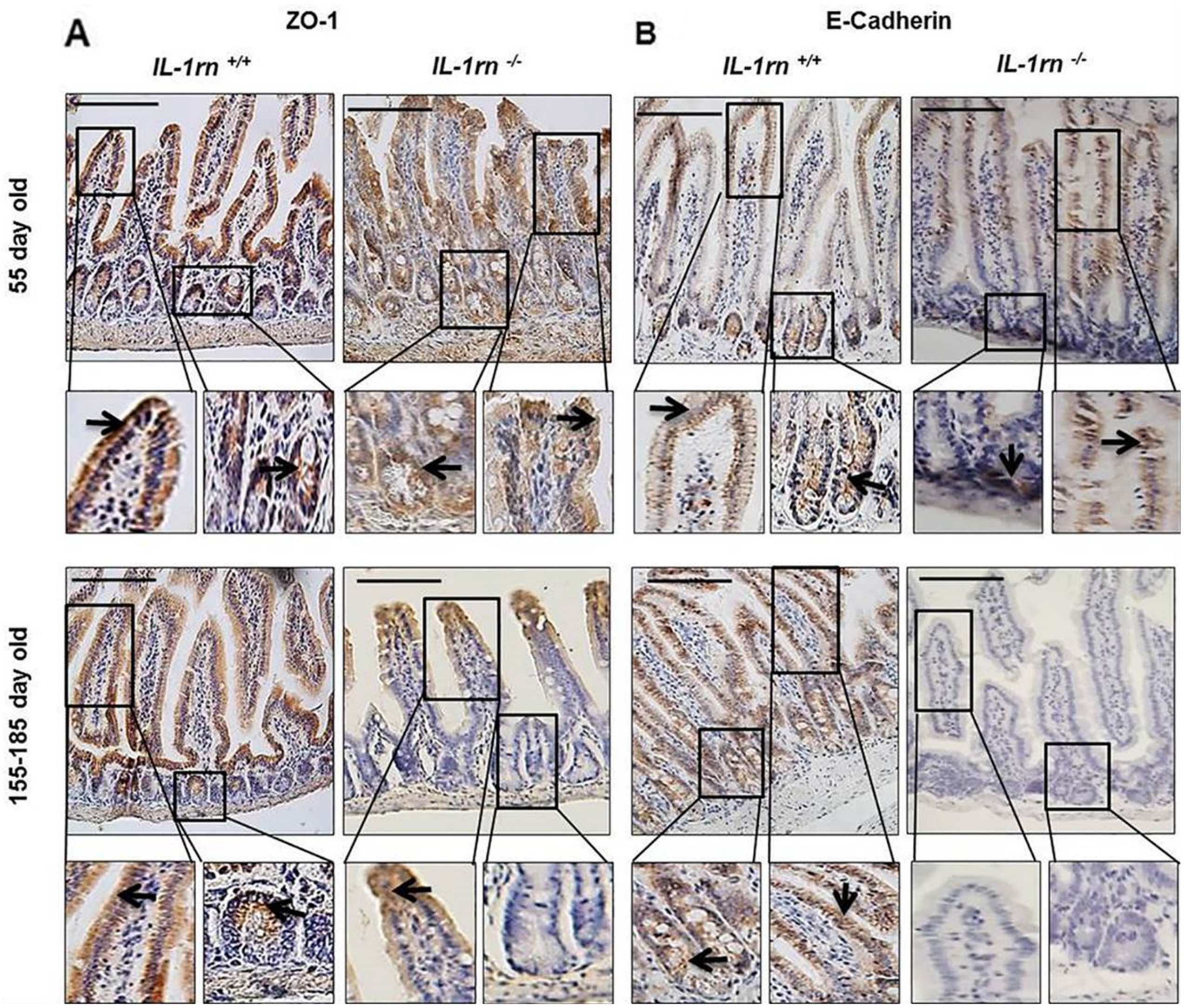

Supplementary Figure 5: Immunohistochemistry staining of the expression and localisation of junctional proteins. (A) ZO-1; **(B)** E-cadherin in the jejunum of the 55-day old and 155-185 day old IL-1rn$^{-/-}$ mice compared with IL-1rn$^{+/+}$ mice. Cell nuclei were stained with haematoxylin (blue). Black arrows indicate immunopositivity. Scale bar = 100μm.

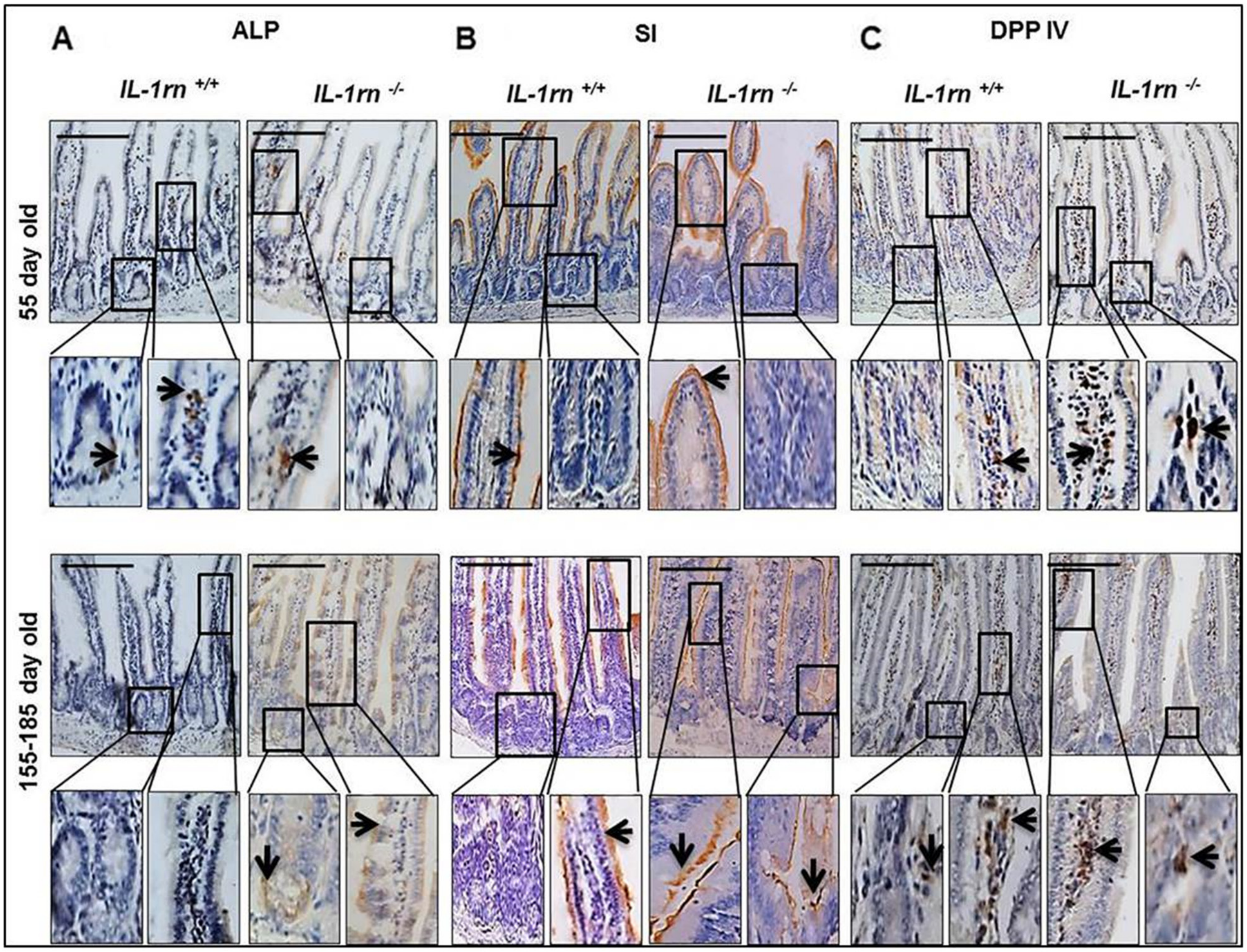

Supplementary Figure 6: Immunohistochemistry staining of the expression and localisation of digestive enzymes. (A) ALP; **(B)** SI and **(C)** DPP IV in the jejunum of the 55-day old and 155-185 day old IL-1rn$^{-/-}$ mice compared with IL-1rn$^{+/+}$ mice. Cell nuclei were stained with haematoxylin (blue). Black arrows indicate immunopositivity. Scale bar = 100µm.
